# The digital trap in the quest for perfect parenting: the relationship between parenting perfectionism and cyberchondria

**DOI:** 10.1186/s40359-026-05174-1

**Published:** 2026-07-22

**Authors:** Büşra Ergin, Esra Ergin, Mehmet Ali Eryılmaz, Abdullah Sami Maden

**Affiliations:** 1 Faculty of Education, Selçuk University, Konya, Türkiye; 2 University of Health Sciences Hamidiye Faculty of Medicine, Konya Health Practice and Research Center, Konya, Türkiye; 3 Department of General Surgery, Konya City Hospital, Konya, Türkiye

**Keywords:** Cyberchondria, Digitalization, Health Anxiety, Online Health Searches, Parenting, Parental Perfectionism

## Abstract

**Background:**

Cyberchondria, characterized by excessive online health-related information seeking accompanied by increased health anxiety, has been associated with various psychological factors. However, research examining its relationship with parenting-specific characteristics remains limited. This study aimed to examine the association between cyberchondria severity and parental perfectionism and to evaluate the role of socio-demographic variables in this relationship.

**Methods:**

This quantitative study was conducted using a cross-sectional correlational survey design. Data were collected from 475 mothers with children in early childhood using the Cyberchondria Severity Scale (CSS), the Multidimensional Parental Perfectionism Scale (MPPS), and a sociodemographic questionnaire. Correlation, regression, and moderation analyses were performed. Moderation effects were tested using the Hayes PROCESS macro (Model 1).

**Results:**

The findings revealed a significant positive correlation between cyberchondria severity and parental perfectionism. Regression analyses indicated that parental perfectionism was a significant predictor of cyberchondria severity, accounting for a substantial proportion of the variance in the model (R^2^ = 0.573). Higher levels of education, employment, income, and the absence of chronic illness were significantly associated with parental perfectionism, whereas age was not significantly associated. Moderation analyses showed that education and income levels significantly strengthened the association between cyberchondria severity and parental perfectionism, indicating that this relationship was more pronounced among individuals with higher socioeconomic status. Employment status and chronic illness did not demonstrate significant moderating effects.

**Conclusions:**

The findings suggest that greater cyberchondria severity is associated with higher levels of parental perfectionism and that this association varies according to socioeconomic characteristics. In particular, education and income appear to strengthen the relationship between cyberchondria severity and parental perfectionism. These results highlight the importance of considering socioeconomic factors when examining the interplay between online health-information seeking behaviors and parenting-related psychological characteristics.

## Introduction

In the process of shaping contemporary family roles, the internet and digital technologies play a decisive role. In this context, digitalization causes a comprehensive transformation by affecting the family institution both at the individual level and at the social, economic, and cultural levels [1]. When examining the historical past of human civilization, it is reported that few revolutions have profoundly affected the structure of social and societal relationships as much as the digital transformation experienced in the twenty-first century. In this regard, digitalization today plays a key role in redefining social bonds such as attachment, participation, and trust, which form the basis of family ties [2].

Digital technologies have brought about a transformation in parenting by influencing the ways in which parents fulfil their child-rearing roles, particularly by reinforcing intensive parenting attitudes [3]. In this context, intensive parenting is a view that holds that a mother must devote all her energy, time and financial resources, as well as her entire emotional capacity, to her child [4]. Another perspective on this view is that the traditional definition of motherhood still persists today and reinforces the image of the “good mother”, thereby fostering perfectionist parenting attitudes [5]. Consequently, the belief that perfectionist parenting leads to good parenting and, in turn, the raising of “good” children compels families to devote a significant amount of time, money and energy to ensuring their children are perfect [6]. These unrealistic expectations placed on children by parents are also regarded as a significant factor in the rise of parental perfectionism. Indeed, the concept of perfectionism, which forms a key pillar of this article, is defined in the literature as an individual setting unrealistically high standards, adhering to these standards, and evaluating their self-worth through them [7]. Parental perfectionism, on the other hand, refers to parents holding themselves to high standards in their parenting [8]. Viewed in this context, the integration of digital technology into parenting practices has paved the way for the emergence of behavioural patterns associated with parental perfectionism.

Studies conducted on adults have determined that highly maladaptive perfectionists carry a relatively higher risk of premature death and chronic illness, struggle to cope with chronic illnesses, and have low levels of individual health control [9]. However, when examining the long-term longitudinal effects of childhood perfectionism, it has been determined that children experience chronic pain in later years and that this pain disrupts daily life activities such as school attendance and participation in socialization [10, 11]. This unhealthy form of perfectionism has also been associated with emotional difficulties accompanied by infections, intestinal diseases, and physical health problems in individuals [12]. Perfectionism, especially when observed during childhood, can lead to internalized problems such as anxiety, depression, and stress, or externalized problems such as anger and aggression in children. Furthermore, decreased sensitivity to mistakes in early childhood causes impairments in emotional regulation skills. These emotional and behavioral self-regulation impairments play an exacerbating role in increasing the risk of suicide, the tendency to engage in crime, or substance use in later years [13]. At this point, as proposed in Family Functioning Theory, a functional family environment is considered important for the development of family members as physically, psychologically, and socially healthy individuals. This is because a functional family is a family model that provides a protective psychosocial foundation where secure attachment is established, supporting the well-being of its members. Conversely, disruptions in family functioning can increase mental health problems and lead to the emergence of anxiety-focused behavior patterns such as cyberchondria [14]. Perfectionism is a personality trait frequently observed in individuals with cyberchondria and is considered a significant risk factor. Xu et al. [15] determined in their studies that perfectionistic individuals, who tend to strive to do their work in the best possible way, also feel the need to interpret disease symptoms flawlessly and completely. Therefore, perfectionism reinforces cyberchondria, compelling these individuals to seek the most consistent and complete explanation for their physical satisfaction. Cyberchondria has been studied more in health-related research [16, 17], and its relationship with parental perfectionism has been addressed very little in studies. In this study, examining the relationship between cyberchondria and parental perfectionism in the context of parents with preschool-aged children will provide a unique contribution to the literature [18].

### Perfectionism and parental perfectionism: theoretical foundations

Perfectionism is conceptualised as a multidimensional personality trait encompassing both harmonious and disharmonious dimensions. It is also defined as a psychological tendency to strive for flawlessness in oneself and/or others, and to set and adhere to extremely high standards [7]. Perfectionist individuals set high standards for themselves, experience a fear of failure, doubt almost all of their actions, and tend to engage in constant self-criticism [19]. Consequently, they make constant efforts to achieve a goal and exhibit traits such as avoiding flaws or shortcomings [20, 21]. Furthermore, from a psychological perspective, it has been suggested that perfectionism is associated with an obsessive personality trait arising from high expectations of achievement and behaviour, and that these individuals possess a rigid superego [22].

In this context, perfectionism is regarded not merely as a behavioural tendency, but as a multi-layered psychological construct linked to deeper, structural personality traits. In the model developed by Hewitt and Flett [7], perfectionism is conceptualised in three dimensions: self-directed, other-directed and externally prescribed perfectionism. In self-directed perfectionism, the individual sets extremely high and often unrealistic standards for themselves and tends to strive for flawlessness in every aspect of their performance. In other-oriented perfectionism, which represents the interpersonal dimension of perfectionism, the individual holds unrealistic expectations of those around them and demands that these people behave in accordance with the high standards they have set. In externally-imposed perfectionism, the individual believes that significant others in their environment impose extremely high standards upon them and feels they must meet these expectations to gain their approval and acceptance. This dimension is also associated with a fear of negative social evaluation and learned helplessness, and reflects the individual’s sensitivity to external evaluations and expectations [7]. This multidimensional nature of perfectionism demonstrates that it is a complex construct arising from the interplay between internal processes and the social context, going beyond individual-level characteristics. Consequently, within the framework of Hewitt and Flett’s model, this study elaborates on the theoretical underpinnings of the concept of perfectionism by drawing on the theoretical approaches of Freud, Adler and Horney—as also proposed by Hewitt et al. (2003)—in order to examine the emergence of perfectionism and its developmental effects on individuals more comprehensively.

When perfectionism is assessed in the light of Freud’s structural theory of personality and his theory of instincts, it is conceptualised not so much as a fundamental internal ‘orientation towards development’ but rather as a structure emerging within the context of psychological conflict and the tendency towards instinctual repetition. According to Freud’s structural theory of personality, the ego functions as a structure that seeks to strike a balance between, on the one hand, the libidinal and aggressive drives originating from the id and, on the other hand, the superego’s rigid moral rules and idealised expectations. Within the context of this structural conflict, the superego, as the representative of internalised moral standards and parental prohibitions, can impose rigid and idealised expectations on the individual [23]. However, Freud does not regard perfectionism as an innate ‘drive for perfection’ that propels human development forward; on the contrary, he argues that at the root of all behaviour lies the tendency of instincts to repeat previous states. Within this framework, development is explained not as a linear progression or an ideal process of perfection, but through the reproduction and repetition of previously experienced states. According to Freud, the fundamental orientation of instincts is the tendency to re-establish a previous state, and this dynamic of repetition determines both life processes and psychological organisation [24, 25]. For this reason, tendencies observed as perfectionism are regarded not so much as a drive towards progress, but rather as an indirect expression of repressed instincts and the drive to repeat. In this process, the ego, interacting with the superego’s rigid and punitive demands, constantly strives to keep the individual’s behaviour under control; as a result, a psychological pattern characterised by intense feelings of guilt, anxiety and self-criticism emerges. In this respect, Freud’s approach is closely related to the self-directed perfectionism dimension in Hewitt and Flett’s [7] model of perfectionism. This is because self-directed perfectionism is characterised by the individual setting extremely high standards for themselves, displaying excessive sensitivity to making mistakes, and constantly evaluating their own performance. Consequently, Freud’s concept of the superego provides an important theoretical foundation for explaining the self-control, self-criticism and pursuit of perfection that underlie self-directed perfectionism.

From the perspective of Adler’s theory of individual psychology, however, he emphasises that social and interpersonal factors, rather than biological drives, are decisive in the development of personality, and in this respect, his approach differs from the Freudian one. According to Adler, understanding an individual requires taking into account the socio-cultural context in which they find themselves and the relationships they form with other people. Within this framework, the human being is regarded as a social being by nature, a structure shaped by social relationships [26]. However, “social interest” is not considered an innate trait, but rather a capacity developed during early childhood through environmental interactions and, in particular, parental attitudes. Within this developmental framework, Adler argues that parental attitudes experienced during childhood play a decisive role in an individual’s capacity for social adaptation and the organisation of their personality. This interactive foundation, combined with the feelings of inadequacy and inferiority experienced by the individual in the early stages of life, shapes their fundamental motivational structure. According to Adler, the “strive for superiority” that emerges at this point is not merely a general tendency towards self-improvement, but a constant psychological orientation aimed at compensating for perceived inadequacies [27]. In this context, perfectionism can be conceptualised as a more rigid, extreme and dysfunctional form of this drive for superiority. In an attempt to overcome feelings of inadequacy and achieve a sense of competence, the individual sets increasingly high and often unrealistic standards for themselves; over time, this can develop into a perfectionist pattern characterised by a constant pursuit of success and a sense of dissatisfaction. Consequently, from this perspective, perfectionism is viewed as a product of the interaction between early-life experiences, the development of social interest and the drive for superiority [28]. In this respect, the Adlerian approach is closely related to the ‘perfectionism towards others’ dimension featured in Hewitt and Flett’s [7] multidimensional model of perfectionism. This is because this dimension is characterised by an individual developing expectations of flawlessness from those around them and, should these expectations not be met, displaying critical and controlling attitudes. Consequently, Adler’s emphasis on the social context and interpersonal interaction demonstrates that perfectionism is not merely an internal trait, but also a construct shaped by relational and environmental expectations.

Within the framework of Horney’s interpersonal (neo-psychoanalytic) theory, perfectionism is regarded as a neurotic tendency stemming from the individual’s underlying experiences of insecurity and anxiety. Within this framework, when an individual feels worthless and inadequate, they develop an idealised self in order to compensate for this negative self-perception and experience an intense sense of compulsion to achieve this ideal. In this process, perfectionism is not limited merely to the individual setting high standards for themselves; it also leads them to generate unrealistic expectations of those around them, thereby transforming into a psychological pattern fuelled by the need for acceptance, approval and a sense of worth. In this context, Horney regards perfectionism as a neurotic tendency linked to the individual’s underlying feelings of insecurity and anxiety [29, 30]. According to Horney [31], when an individual feels inadequate and worthless, they develop an idealised self in order to cope with these feelings and make intense efforts to live up to this ideal. This process forms the basis of what Horney termed the “quest for grandeu”. Within this quest for grandeur, the need for perfection in particular leads the individual to set unrealistic and unattainable standards for themselves. Consequently, whilst constantly striving to be better and flawless, the individual may overlook their own limitations and experience a chronic sense of inadequacy. Horney also argues that the individual bases their self-worth largely on external approval and acceptance; consequently, they may experience intense disappointment and a sense of worthlessness in the face of failure or criticism. Within this framework, perfectionism is regarded as a neurotic compulsion aimed at maintaining an idealised self-image and safeguarding external approval. Perfectionism, as examined within the framework of Horney’s interpersonal theory, is directly related to the ‘other-determined perfectionism’ dimension in Hewitt and Flett’s [7] multidimensional model of perfectionism, particularly because the individual bases their self-worth on external approval and social evaluations. This dimension is characterised by an individual’s perception of expectations of perfection from their environment and the development of intense anxiety, feelings of inadequacy and self-criticism when they fail to meet these expectations. Consequently, Horney’s concepts of the idealised self and the quest for grandeur provide a theoretical foundation for explaining the structure of perfectionism, which is shaped by external validation and interpersonal pressure. Consequently, the theoretical approaches of Freud, Adler and Horney offer a complementary framework that addresses perfectionism at different levels of psychological analysis; when considered alongside Hewitt and Flett’s [7] multidimensional model, it becomes clear that perfectionism is not limited solely to an individual’s self-imposed standards, but rather exhibits a multi-layered structure shaped by the interaction between intrapsychic processes, interpersonal relationships and social expectations.

Nowadays, the perfectionism expected of almost every member of society creates increasing pressure to perform and a constant need to meet high standards, thereby paving the way for the emergence of various psychological problems such as depression, stress, anxiety and eating disorders. Consequently, studies on perfectionism frequently examine its relationship with these variables and analyse perfectionism in terms of various psychological outcomes [32–37]. However, it is increasingly recognised that perfectionism is not limited solely to individual outcomes, but also has significant implications for the family system and parenting processes. Accordingly, in recent years, researchers have shown increasing interest in parental perfectionism, which is the manifestation of perfectionism within the parenting context [38–41].

As is frequently emphasised in research on the family, the family environment constitutes one of the most important socio-environmental contexts for a child’s development. The main reason for this is that parents serve as a constantly accessible resource for their child and exert a long-term and lasting influence on their developmental processes [42, 43]. This influence is linked not only to the supportive and protective aspects of parenting but also to the potential for parents to shape a child’s psychological functioning through their attitudes and expectations. A key concept in this context is parental perfectionism, which is defined as parents’ expectations that their children will fulfil the ideals and aspirations that they themselves have been unable to achieve, alongside their critical attitudes and judgements regarding the children’s failure to meet these expectations [44].

Research conducted in recent years has highlighted the impact of parental perfectionism on parenting processes, distinguishing between two main forms: parenting perfectionism and child-focused perfectionism [45–47]. Whilst parental perfectionism is characterised by parents setting high standards for themselves and experiencing intense anxiety about making mistakes, child-focused perfectionism involves expectations of high performance, order and flawless behaviour from children [48]. These high standards and expectations lead parents to constantly evaluate both their own behaviour and their children’s performance, and over time this can create a significant psychological burden [49]. If these expectations are not met, parents may perceive this as a personal failure; this situation not only increases parent–child conflict but can also have negative effects on parental self-efficacy and psychological well-being [50].

In the literature, parental perfectionism is predominantly explained through various theoretical frameworks focusing on its effects on child development. According to the dual-process model proposed by Slade and Owens [25]—one such framework—perfectionism is not a single construct; it can manifest in two distinct functional forms: “positive/adaptive perfectionism”, which aims to achieve success, and “negative/maladaptive perfectionism”, which focuses on avoiding failure. According to this model, individuals with high levels of positive perfectionism are able to accept their own limitations more realistically and do not base their self-worth solely on performance outcomes [25, 51]. Consequently, parents with this type of perfectionism are more likely to adopt a parenting style that is warmer, more supportive and characterised by unconditional acceptance towards their children. In contrast, negative perfectionism is characterised by critical self-evaluation, excessive sensitivity to making mistakes and a constant feeling of inadequacy [22]. Parents with these characteristics may develop more critical attitudes towards both themselves and their children, and may tend to constantly evaluate their children’s behaviour. Indeed, research shows that negative perfectionism is associated with psychological control and overly controlling attitudes in parents [52, 53]. Negative perfectionism is also highlighted in the literature as a risk factor that negatively affects the development of emotional intelligence in children and can pave the way for the emergence of psychological patterns such as self-denial, helplessness, feelings of inferiority and depressive mood [54–56]. Furthermore, these individuals’ high sensitivity to criticism and negative feedback paves the way for them to develop a more anxious, controlling and error-focused approach in their parenting. From this perspective, it is evident that parental perfectionism should be regarded not merely as a trait passed on to the child, but also as a significant psychological determinant shaping parenting behaviours.

In line with this theoretical framework, a review of the literature on parental perfectionism reveals that, while the concepts outlined in the table are conceptually interrelated, they represent different dimensions of the contemporary parenting experience. These concepts contribute to the theoretical understanding of parental perfectionism and explain the individual, social, and cultural processes that may influence the emergence of this construct. However, the primary focus of this study is not the concepts themselves, but rather the examination of the relationship between parental perfectionism and cyberchondria. For this reason, the relevant concepts were treated as contextual structures supporting the conceptual framework of parental perfectionism, but were not considered as primary variables of the study (Table 1).

**Table 1:** Conceptual comparison of the core constructs related to parenting perfectionism

Construct	Core definition	Primary focus	Key characteristic	Relation to present study
Intensive Parenting [3, 6, 13]	A child-centered parenting ideology emphasizing high parental involvement and investment	Child development and success	Extensive parental responsibility	Provides the cultural context in which perfectionistic parenting standards may emerge
Ideal Motherhood [5, 48]	Socially constructed beliefs regarding the characteristics of a “good mother”	Maternal identity	Unrealistically high maternal standards	May contribute to internalized parenting expectations
Socially Imposed Parenting Expectations [7, 36]	Perceived pressure from society to meet parenting standards	External evaluation	Social comparison and pressure	May reinforce perfectionistic parenting tendencies
Parenting Anxiety [30, 33, 35, 39, 54, 55]	Worry and concern regarding parenting performance and child outcomes	Emotional response	Fear of making mistakes	May accompany perfectionistic parenting beliefs
Parental Perfectionism [44–46]	The tendency to set excessively high standards for parenting and evaluate oneself critically as a parent	Parenting performance	Perfectionistic self-evaluation and concern over mistakes	Main predictor examined in the present study

Although examining parental perfectionism within the framework of the dual-process model simplifies the concept of perfectionism from a conceptual perspective, it is evident from the literature that this construct is also explained by more comprehensive and multidimensional models. Indeed, in Hewitt and Flett’s [7] multidimensional model of perfectionism, parental perfectionism is assessed in the context of the parenting role, taking into account its self-directed, other-directed and externally determined dimensions. In this regard, the parent’s high standards for themselves, their expectations of the child, and the child’s perception of the parent’s expectations are regarded as the fundamental mechanisms that bring the interpersonal nature of perfectionism into the parenting process. Furthermore, in the literature, perfectionism is approached not merely as a fixed personality trait, but also as a dynamic construct shaped through cognitive schemas, learned evaluation patterns and social learning processes [57–60]. Consequently, whilst the dual-process model provides an explanatory and operational foundation for understanding the effects of parental perfectionism, its evaluation in conjunction with multidimensional and process-based approaches offers a more holistic theoretical perspective.

### Cyberchondria: conceptual development and digital context

The term “cyberchondria” is derived from the words “cyber” and “hypochondriasis” and refers to a form of hypochondriasis linked to or associated with the use of the internet or computers [61]. However, whilst it is not entirely clear who first coined the term or when it was first used, it is noted that it was initially used by the media to describe the negative aspects of digital health [61, 62]. Some sources on the subject, however, argue that the term originated from an article titled “Illnesses are rife on the internet; fuelling the anxieties of hypochondriacs”, written by journalist Ann Carrns in the Wall Street Journal in 1999 [61].

The media subsequently adopted the term “cyberchondria”, helping to popularise the concept due to its novelty and its association with technology. In this context, cyberchondria is defined as a new risk emerging in the field of health-related internet use, characterised by excessive or repetitive health-related searches online that cause anxiety and distress. Cyberchondria is generally described as a dangerous and uncontrolled product of modern society; it is characterised as “rapidly spreading (going viral)” and a “destructive obsession”. The view that this concept defines our times was further supported by its selection as one of the “words of the year” by Webster’s New World Dictionary in 2008, whilst some studies have referred to the current era as the “age of cyberchondria” [63, 64]. This situation has led to the concept frequently being discussed in the academic literature on the basis of media sources and being used with different meanings in different contexts. In relation to this concept, the transformation of individuals’ online information-seeking behaviour—whether for themselves or their loved ones—into cyberchondria behaviour has led to these individuals being referred to in popular parlance as ‘Dr Google’ [65], as the internet provides them with a means of self-help and self-management. Subsequently, as researchers took a keen interest in the concept, various definitions of cyberchondria were developed. Cyberchondria is defined as a term used to describe individuals’ excessive online search behaviour aimed at accessing medical information as a result of increased health anxiety [66]. In addition to this definition, definitions of cyberchondria converge on two key points: repetitive/excessive online health information-seeking behaviour and increased health anxiety [67]. The most widely accepted definition was provided by Starcevic and Berle [61]. According to this definition, cyberchondria is excessive or repetitive behaviour involving the search for health information online, triggered by health-related anxiety or distress, which, rather than alleviating this anxiety, actually exacerbates it. This approach distinguishes cyberchondria from normal health information-seeking behaviour and treats it as a syndrome-like construct comprising a cluster of interrelated symptoms [68].

The conceptual prevalence of cyberchondria is thought to stem, in particular, from the fact that, nowadays, an increasing number of people are turning to the internet for answers to their health-related questions. In this regard, a survey conducted in 2010 as part of a US study found that 88 per cent of adult internet users had searched for health information online at least once, whilst 62 per cent had done so within the past month [69]. Furthermore, a study involving over 12,000 participants across 12 countries revealed that in nine of these countries—including Russia, China, India, Mexico, Brazil, the US, Italy, Australia and Germany—more than 75% of participants had engaged in health-related information-seeking behaviour [70]. In the Turkish context, according to data from the Turkish Statistical Institute [71], the internet usage rate among the 16–74 age group rose from 88.8% in 2024 to 90.9% in 2025. This increase demonstrates that the internet has become an almost universal component of daily life and has established itself as a fundamental tool, particularly in terms of access to information, communication and health-related information-seeking behaviour. Whilst this widespread adoption of the internet facilitates access to health-related content, it simultaneously increases the risk of exposure to incorrect, incomplete or unverified information, thereby creating a fertile ground for dysfunctional health information-seeking patterns such as cyberchondria. Indeed, data from TÜİK [71] also show that individuals widely use the internet to obtain health-related information. It was found that 63.3% of individuals who had used the internet in the past three months had searched for information online on topics such as injuries, illnesses, nutrition and general health improvement; the proportion searching for information on physical health stood at 60.9%, whilst the proportion searching for information on mental health remained at 28.4%.

With the onset of the COVID-19 pandemic, however, the internet and social media have become a significant global source of health information thanks to the rapid flow of information [72]. These platforms have facilitated an understanding of the severity of the pandemic, whilst also increasing anxiety and thereby contributing to the adoption of protective behaviours such as hygiene, social distancing, quarantine and mask-wearing [73]. However, the rise in anxiety and uncertainty during the pandemic, coupled with easier access to social media, has also accelerated the emergence of cyberchondria, defined as a compulsive search for information [74, 75].

A review of studies on cyberchondria reveals that a non-clinical study conducted by White and Horvitz [76]found that over 40% of individuals who frequently searched for health information online experienced increased health anxiety. Furthermore, various studies indicate that cyberchondria is associated with adverse outcomes such as functional impairment [77], a decline in quality of life [78] and lower satisfaction with consultations with doctors [79]. Therefore, from a treatment perspective, a more detailed and multidimensional approach is required to assess this health-related phenomenon, which is becoming increasingly prevalent in the digital age, more accurately [66]. These findings demonstrate that the internet has become a widespread and central source of health information-seeking behaviour. However, the tendency for individuals to repeatedly turn to unverified online information in situations of health-related uncertainty can trigger the excessive and uncontrolled cycle of health information seeking, which is one of the key characteristics of cyberchondria. In this context, the widespread use of the internet can be regarded not merely as a factor that facilitates access to information, but also as a risk factor that paves the way for the emergence of cognitive and behavioural processes that perpetuate health-related anxiety.

### Cyberchondria and parenting perfectionism: research gap, aim, and hypotheses

Although studies on psychological and behavioural variables associated with cyberchondria have increased in recent years, research examining the relationship between parenting-specific characteristics and cyberchondria remains limited. Parenting is a process in which responsibilities relating to a child’s health and development are intensely experienced and which frequently requires health-related decisions to be made. Consequently, parents frequently turn to online resources to obtain information about their children’s health. For parents of young children in particular, the internet is viewed as a key resource providing quick and easy access to health-related information [80]. However, this ease of access does not always reduce health-related uncertainty; it can lead to increased anxiety in some parents and cause their online health information-seeking behaviour to develop into a repetitive and dysfunctional pattern. This situation, in turn, may contribute to the emergence of cyberchondria behaviours in parents [81–83].

Studies conducted within the parenting context have identified that parental cyberchondria is associated with parental stress [84], parental digital literacy [85, 86]and parents’ health concerns regarding their child [87]. However, it is evident that the number of studies examining the relationship between parenting and cyberchondria is limited [88] and that research addressing the role of psychological characteristics related specifically to parenting in cyberchondria remains insufficient [89]. In this context, parental perfectionism can be regarded as an important yet under-researched variable that may influence parents’ responses to health-related uncertainties and their online health information-seeking behaviour. Consequently, examining the relationship between parental perfectionism and cyberchondria will contribute to a better understanding of the psychological processes underlying digital health anxiety in parents.

It is known that various psychological and behavioural factors play a role in the development of cyberchondria in individuals [85]. In this context, personality traits such as neuroticism, anxiety sensitivity and perfectionism are cited as significant predictors of cyberchondria [90]. Frost and Shows [91] explain the relationship between perfectionism and indecisiveness in terms of individuals’ excessive focus on mistakes and their constant questioning of their performance. When considered in the context of parenting, this situation bears similarities to the nature of online health-related information-seeking behaviour. The constant search for reassurance regarding health can lead individuals to enter repetitive cycles of information-seeking in an attempt to reduce uncertainty, thereby reinforcing cyberchondria behaviours [61]. Contradictory and often unverified health information, particularly that encountered online, can heighten parents’ perception of risk regarding their children’s health, thereby reinforcing the need for constant monitoring, supervision and making the most appropriate decisions. This process can pave the way for parents to adopt higher standards regarding their children’s care and development, to avoid making mistakes, and to increase their efforts to fulfil their parenting roles flawlessly. Indeed, it has been noted that the need for control associated with intensive and repetitive search behaviour regarding online health information is linked to cognitive processes related to perfectionism, such as intolerance of uncertainty and the search for reassurance [92]. In this regard, cyberchondria can be regarded not merely as a consequence of perfectionism, but also as a potential precursor variable that may contribute to parents developing standards of flawless parenting.

**Fig. 1:**
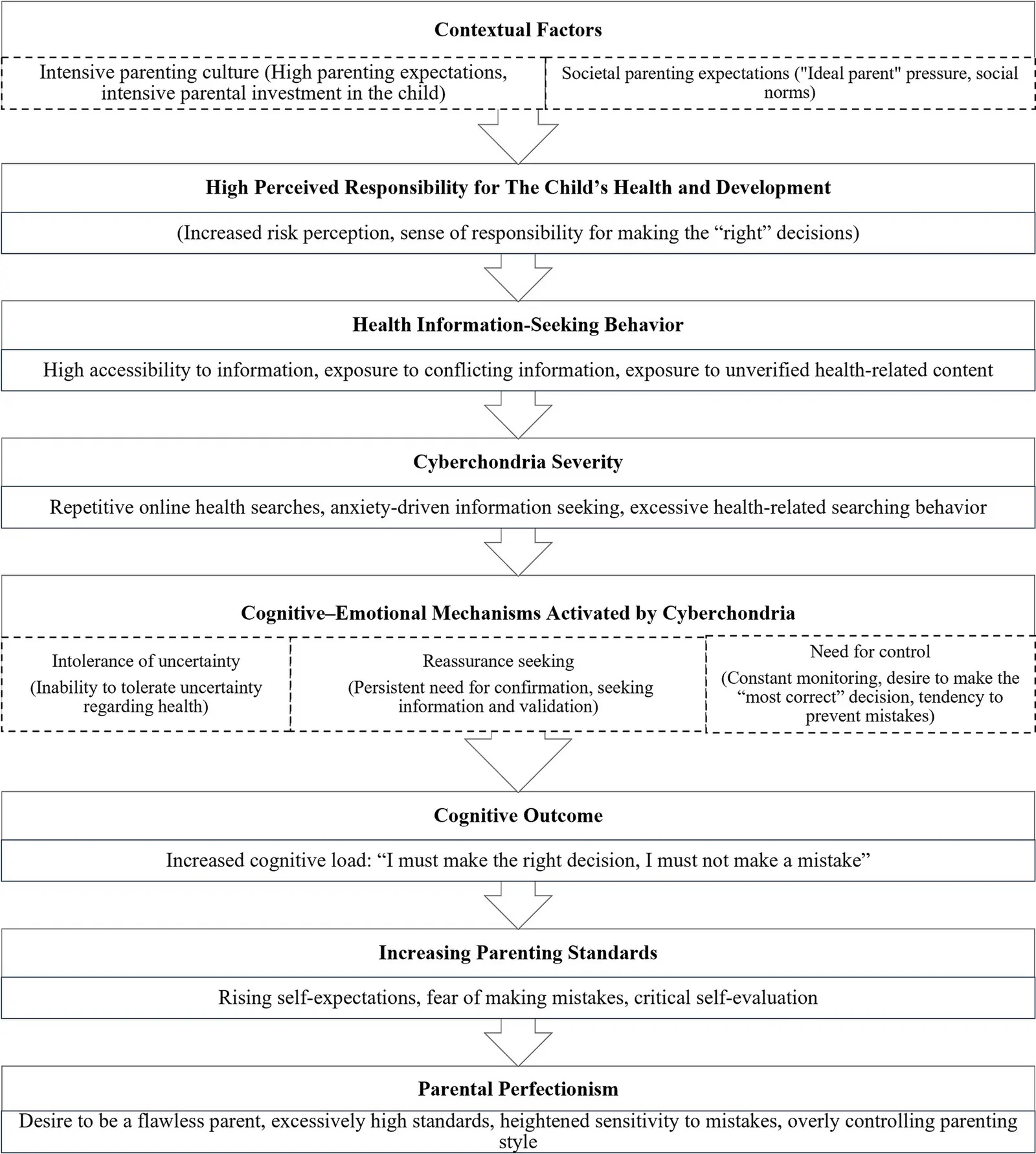
Conceptual model of the relationship between cyberchondria and parental perfectionism

However, just as it is assumed that situational information-seeking behaviours such as cyberchondria stem directly from personality traits, it should not be overlooked that these behaviours may, over time, shape higher-order tendencies by reinforcing cognitive and emotional processes. In particular, mechanisms such as intolerance of uncertainty, the need for control and the search for reassurance can be continuously activated through repetitive online health information-seeking behaviour, and this process can reshape individuals’ norm-setting tendencies. Viewed from this perspective, cyberchondria may function not only as a reflection of an existing level of perfectionism, but also as a process variable that contributes to the adoption of higher parenting standards by increasing the pursuit of control and competence within the parenting context. Particularly in situations involving high levels of responsibility and perceived risk, such as child health, it is thought that the fragmented and often contradictory content obtained through online information-seeking [93, 94] increases the cognitive pressure on parents to “make the right decision”. This situation may pave the way for the emergence, over time, of stricter, higher and more error-intolerant parenting standards. In this context, Fig. 1 presents a conceptual model illustrating the proposed relationship between cyberchondria and parental perfectionism. The model suggests that contextual factors reflecting the contemporary parenting environment (intense parenting culture and societal parenting expectations) reinforce parents’ sense of responsibility for their children’s health and development, thereby supporting online health information seeking behavior and cyberchondria. Furthermore, the model proposes that cyberchondria increases parental cognitive pressure and parenting standards by activating cognitive-emotional mechanisms such as intolerance of uncertainty, reassurance seeking, and the need for control, thus contributing to parental perfectionism. Consequently, contrary to the prevailing assumption in the literature, modelling cyberchondria as a predictor of parental perfectionism is theoretically justifiable and warrants further investigation. To this end, this study examined the relationship between the severity of cyberchondria and parental perfectionism among mothers with young children.

#### Research hypotheses

In this study, the following hypotheses will be examined based on Hewitt and Flett’s model of perfectionism and existing empirical evidence.

- H1. There is a significant and positive relationship between mothers’ levels of cyberchondria and their parental perfectionism.
- H2. Cyberchondria provides a meaningful and positive explanation for parental perfectionism.
- H3. Demographic variables (age, educational level, employment status, income level, chronic illness) significantly explain parental perfectionism.
- H4. Demographic variables (level of education, employment status, income level, chronic illness) significantly moderate the relationship between the severity of cyberchondria and parental perfectionism.

## Method

### Research model

This study was designed as a cross-sectional study based on a correlational survey model within a quantitative research framework. The primary aim of the study is to examine the relationship between the severity of cyberchondria and parental perfectionism, and to assess the role of socio-demographic variables in this relationship. The independent variable in the study is the severity of cyberchondria (X). Cyberchondria is defined as a form of anxiety characterised by individuals’ excessive and repetitive searching for health-related information on online platforms, spending a considerable amount of time doing so, comparing the information obtained with the views of healthcare professionals, and experiencing negative emotional reactions as a result of health information found on the internet. The Cyberchondria Severity Scale (CSS) was used to assess this condition. The dependent variable in the study is parental perfectionism (Y). Parental perfectionism is defined as parents setting high standards for their child-rearing practices, showing a tendency to avoid making mistakes, and feeling intense cognitive and emotional pressure to be the ideal parent. The Multidimensional Parenting Perfectionism Scale (MPPS) was used to measure this variable. The study also examined the relationship between the severity of cyberchondria and parenting perfectionism, whilst also testing the predictive effect of the severity of cyberchondria on parenting perfectionism. In addition, the moderating roles of sociodemographic variables—such as educational attainment, employment status, income level and the presence of chronic illness—as well as their explanatory effects on parental perfectionism were analysed. The age variable, meanwhile, was treated as a control variable in the model. Sociodemographic data were collected to determine the participants’ demographic characteristics and to be used as a control variable in group comparisons and regression analyses. Accordingly, the research is based on a comprehensive model that examines the relationships between variables, predictive effects and partial moderating mechanisms.

### Participants

The sample size for the study was determined using the G*Power 3.1.9.4 software. In the sample size calculation, the correlation coefficient (|ρ|= 0.165) reported in the study by Teken et al. [95] (2026), which examined the relationship between the severity of cyberchondria and health literacy, was used as a basis [96]. According to the a priori power analysis carried out, it was determined that at least 467 participants were required, assuming 95 per cent statistical power (power = 0.95) and a 5 per cent significance level (α = 0.05). Taking into account potential data losses and missing responses, approximately 500 participants were reached. As a result of the data cleaning process, forms containing missing or incorrect data were excluded, and the analyses were carried out on a sample of 475 participants.

The study was carried out using a research-based sampling method. The inclusion criteria for participants in the study were: (i) voluntary participation in the study, (ii) having a child in early childhood (0–8 years), (iii) being aged between 25 and 50, (iv) having a sufficient command of Turkish to understand and respond to the scales, and (v) having no history of any psychiatric diagnosis or active psychiatric treatment. Exclusion criteria are defined as failure to meet the inclusion criteria and the completion of data collection forms in an incomplete, incorrect or inconsistent manner.

The participants’ ages ranged from 25 to 50, with an average age of 35.2 (SD = 5.1). An analysis of the age distribution shows that there were 205 participants under the age of 35 (*n* = 205, 43.2%) and 270 participants aged 35 and over (*n* = 270, 56.8%). In terms of educational attainment, 297 of the participants (*n* = 297, 62.5%) hold a bachelor’s degree, whilst 178 (*n* = 178, 37.5%) have a postgraduate qualification. An analysis of employment status revealed that 408 participants (*n* = 408, 85.9%) were in active employment, whilst 67 participants (*n* = 67, 14.1%) were not in employment. In terms of income level, 304 participants (*n* = 304, 64.0%) fall into the income group of 60,000 TL and above, whilst 171 participants (*n* = 171, 36.0%) have an income level below 60,000 TL. When the distribution of the number of children was analysed, 193 participants (*n* = 193, 40.6%) had one child, 183 participants (*n* = 183, 38.5%) had two children, and 99 participants (*n* = 99, 20.8%) had three or more children. When chronic conditions were examined, 146 participants (*n* = 146, 30.7%) reported having at least one chronic condition (e.g. cancer, migraine, fibromyalgia, asthma, diabetes, eczema, hypertension, thyroid disorders, chronic fatigue, rheumatoid arthritis, FMF, tobacco dependence, ankylosing spondylitis, obesity), whilst 329 participants (*n* = 329, 69.3%) did not report any chronic conditions.

### Data collection tools

Demographic data on the study group were obtained using a personal information form prepared by the researchers.

#### Multidimensional Parenting Perfectionism Scale (MPPS)

The original version was developed by Snell et al. [97], and the Turkish adaptation was carried out by Taluy and Maraş [98]. The measurement instrument assesses multiple components of parenting perfectionism. The Turkish version comprises 23 items, four sub-dimensions and a response system allowing for a five-point rating scale. There are no reverse-scored items. The internal consistency coefficient α for the measurement instrument adapted into Turkish is 0.92, and the internal consistency coefficients for its subscales range from 0.83 to 0.92. For the sample in this study, the calculated internal consistency coefficient α is 0.96, and the internal consistency coefficients for its subscales range from 0.83 to 0.93.

#### Cyberchondria Severity Scale (CSS)

The original version was developed by McElroy and Shevlin [99], and the Turkish adaptation was carried out by Selvi et al. [100]. The measurement tool assesses individuals’ prolonged online health-related searches and their efforts to access information. The Turkish version comprises 33 items, five sub-dimensions and a five-point rating scale. Items 9, 28 and 33 are reverse-scored. The internal consistency coefficient (α) for the assessment tool adapted into Turkish is 0.91, and the internal consistency coefficients for its subscales range from 0.64 to 0.87. For the sample in this study, the calculated internal consistency coefficient (α) is 0.98, and the internal consistency coefficients for its subscales range from 0.74 to 0.97.

### The data collection process and data analysis

During the data collection process, volunteer participants who met the inclusion and exclusion criteria were contacted via face-to-face interviews, online interviews and telephone interviews, and their consent was obtained. Once consent had been obtained, questions regarding the assessment tools to be used in the study were sent to them via Google Forms. The data obtained were checked by the researchers for incomplete, incorrect and inconsistent responses and subsequently cleaned. The average time taken by participants to complete the assessment tools was found to be 20 min.

Data analysis was performed using IBM SPSS Statistics Standard Concurrent User V26 (IBM Corp., Armonk, New York, USA) and AMOS 26 software packages. Descriptive statistics were reported as the sample size (n), percentage (%), mean (X̄), standard deviation (SD), median (M), minimum (min), maximum (max) and interquartile range (IQR). The distribution characteristics of the data were assessed using skewness and kurtosis values, and it was found that these values fell within acceptable limits. However, the results of the Kolmogorov–Smirnov test showed that the data did not satisfy the assumption of a normal distribution. For this reason, non-parametric tests were preferred in the analyses. The Mann–Whitney U test was used for comparisons between two groups, whilst the Kruskal–Wallis H test was used for variables with more than two groups. Where the Kruskal–Wallis H test yielded a significant result, pairwise comparisons were carried out using the Bonferroni correction. The relationship between the severity of cyberchondria and parental perfectionism was examined using Spearman’s rho correlation coefficient. When interpreting correlation coefficients, values of *r* < 0.30 are considered to indicate a low level of correlation, 0.30–0.70 a moderate level, 0.71–0.90 a high level, and above 0.90 a very high level of correlation [101]. The effect of the sociodemographic characteristics found to be significant in univariate analyses on parental perfectionism was assessed using multiple linear regression analysis. Categorical variables have been included in the analyses as dummy variables. Robust standard errors were used in the regression analyses due to the data not following a normal distribution and the possibility of heteroscedasticity. Robust methods enable the calculation of more reliable standard errors, confidence intervals and significance tests by mitigating the effects of violations of regression assumptions [102–104]. The assumptions of the regression models were tested using tolerance and VIF values for multicollinearity, the Durbin–Watson statistic for autocorrelation, and Q-Q plots for the normality of the residuals. Furthermore, Hayes’s PROCESS macro (Model 1) was used to examine regulatory effects, and the analyses were carried out using 5,000 bootstrap samples. This method enables the assessment of direct and interaction effects between variables within the framework of regression-based path analysis [105]. The bootstrap method was chosen to enhance the reliability of the estimates using a resampling approach [106, 107]. In addition, the structural model was created using the AMOS software, and the fit indices for the model were calculated and evaluated during the analysis process. In all analyses, a *p*-value of less than 0.05 was taken as the threshold for statistical significance.

### Research ethics

The procedures followed regarding the ethical aspects of the research were as follows:

- The necessary permissions to use the assessment tools in the research were obtained via email (from Assoc. Prof. Dr Nur TALUY for the MPPS; from Assoc. Prof. Dr Ali KANDEĞER for the CSS).
- As the study group consisted of human participants, the study was deemed ethically acceptable by the Hamidiye Scientific Research Ethics Committee of the University of Health Sciences at its meeting held on 09 January 2025 (Decision No. 1/7), as confirmed by Approval Letter No. 34996 dated 28 January 2025.
- During data collection, participants were informed that the data would be used solely for scientific purposes and that an anonymous evaluation would be carried out. Informed consent was subsequently obtained.
- Compliance was ensured with the Guidelines on Scientific Research and Publication Ethics for Higher Education Institutions; no breaches of the provisions outlined in Sect. "Method", titled “Actions Contrary to Scientific Research and Publication Ethics”, were committed.
- The procedures were conducted in accordance with the principles set out in the Declaration of Helsinki regarding human research.

## Findings

This section contains information regarding the data obtained as a result of the analysis of the study’s parameters.

Table 2 presents the descriptive statistics, measures of normality, reliability coefficients and relationships between variables for the measurement instruments used in the study. The mean score for parenting perfectionism was calculated as 77.49 (SD = 19.87), whilst the median was 74; the observed scores ranged from 36 to 115. The mean score for cyberchondria severity was 100.01 (SD = 37.53), the median was 99, and scores ranged from 38 to 153. The levels of internal consistency of the measurement instruments were assessed using Cronbach’s alpha coefficients. The reliability coefficient calculated for the Parenting Perfectionism Scale is 0.960, whilst that for the Cybersychosis Severity Scale is 0.984. Both coefficients indicate that the measurements demonstrate a very high degree of internal consistency. Upon examining the distribution characteristics, the skewness and kurtosis values for parental perfectionism scores were found to be −0.049 and −0.591, respectively; whilst those for cyberchondria severity scores were −0.145 and −1.488. It was determined that the results fell within an acceptable range (−2, + 2) [108]. According to the results of the Kolmogorov–Smirnov test, it was determined that the MPPS (KS = 0.183; *p* < 0.001) and CSS (KS = 0.170; *p* < 0.001) distributions showed statistically significant differences; however, the data were found not to be normally distributed. For this reason, non-parametric statistical methods were preferred in subsequent analyses. The results of the Spearman rank-difference correlation analysis revealed a positive, moderate and statistically significant relationship between parental perfectionism and the severity of cyberchondria (*r* = 0.663, *p* < 0.001). Accordingly, an increase in the severity of cyberchondria is associated with an increase in parental perfectionism (Table 2).

**Table 2:** Descriptive statistics, normality tests and reliability results for the scales (*n* = 475)

	**Statistics**	**CR**	**Skewness**	**Kurtosis**	**KS**	**MPPS**
MPPS		0.960	−0.049	−0.591	0.183**	**-**
*Mean* ± *SD*	77.49 ± 19.87					
*M* (*min*–*max*)	74 (36–115)					
CSS		0.984	−0.145	−1.488	0.170**	*r* = 0.663**
*Mean* ± *SD*	100.01 ± 37.53					
*M* (*min*–*max*)	99 (38–153)					

*r* Spearman's rank correlation coefficient, *SD* Standard Deviation, *M* Median, *CR* Cronbach Alfa, *KS* Kolmogorov–Smirnov, *MPPS* Multidimensional Parenting Perfectionism Scale, *CSS* Cyberchondria Severity Scale

^**^p < 0.001

Table 3 presents the findings regarding the comparison of scores for parental perfectionism (MPPS) and cyberchondria severity (CSS) according to sociodemographic characteristics. When analysed by age group, it was found that the mean MPPS scores of participants aged 35 and over (X̄ = 78.56, SD = 15.89) were significantly higher than those of participants under 35 (X̄ = 76.09, SD = 24.11) (Z = −1.985, *p* = 0.047). However, no statistically significant difference was found between age groups in terms of CSS scores (Z = −1.595, *p* = 0.111). When educational attainment was examined, it was found that the MPPS scores of participants with postgraduate qualifications (X̄ = 82.69, SD = 18.82) were significantly higher than those of participants with a bachelor’s degree (X̄ = 74.38, SD = 19.87) (Z = −4.272, *p* < 0.001). However, no significant difference was found in CSS scores according to educational level (Z = −1.385, *p* = 0.166). In comparisons based on employment status, it was found that the MPPS scores of employed participants (X̄ = 79.43, SD = 19.38) were significantly higher than those of unemployed participants (X̄ = 65.70, SD = 18.89) (Z = −5.213, *p* < 0.001). As regards CSS scores, however, no significant difference was found based on employment status (Z = −1.838, *p* = 0.066). Income level showed a significant difference in terms of both MPPS and CSS scores. The MPPS scores of participants with a monthly income of 60,000 TL or more (X̄ = 83.97, SD = 18.63) are significantly higher than those of participants with an income below 60,000 TL (X̄ = 65.96, SD = 16.54) (Z = −8.446, *p* < 0.001). Similarly, it was found that CSS scores were significantly higher in the high-income group (X̄ = 110.08, SD = 35.71) than in the low-income group (X̄ = 82.12, SD = 33.93) (Z = −7.457,* p* < 0.001). No significant difference was found in MPPS scores according to the number of children (H = 1.578, *p* = 0.454). In contrast, CSS scores did show a significant difference according to the number of children (H = 9.309, *p* = 0.010). Following Bonferroni-corrected multiple comparisons, it was found that the CSS scores of participants with one child were lower than those of participants with two children and those with three or more children. When assessed in terms of chronic illness status, it was found that the MPPS (X̄ = 82.39, SD = 18.76) and CSS (X̄ = 118.58, SD = 30.61) scores of participants with a chronic illness were significantly higher than those of participants without a chronic illness (X̄ = 75.32, SD = 20.00; X̄ = 91.77, SD = 37.41) (MPPS: Z = −3.285, *p* = 0.001; CSS: Z = 6.510, *p* < 0.001). Overall, the findings indicate that parental perfectionism varies in particular according to age, educational attainment, employment status, income level and the presence of chronic illness; whilst the severity of cyberchondria is more closely associated with income level, number of children and the presence of chronic illness (Table 3).

**Table 3:** Comparison of MPPS and CSS scores according to descriptive characteristics (*n* = 475)

	**Category**	***n***	***%***	MPPS		CSS	
				***Mean* ± *SD***	***M* (*IQR*)**	***Mean* ± *SD***	***M* (*IQR*)**
Age [X̄ ± SD/M(min–max): 35.2 ± 5.1/35 (25–50)]	Under 35	205	43.2	76.09 ± 24.11	71 (41)	102.69 ± 37.39	109 (77)
	Aged 35 and over	270	56.8	78.56 ± 15.89	74 (24)	97.98 ± 37.58	99 (78)
*Z* (*p*)				−1.985 (0.047)*		−1.595 (0.111)	
Level of education	Bachelor’s degree	297	62.5	74.38 ± 19.87	74 (16)	98.71 ± 35.25	99 (57)
	Postgraduate	178	37.5	82.69 ± 18.82	76 (32)	102.19 ± 41.07	123 (89)
*Z* (*p*)				−4.272 (< 0.001)**		−1.385 (0.166)	
Employment status	Working	408	85.9	79.43 ± 19.38	74 (35)	101.51 ± 36.55	109 (70)
	It’s not working	67	14.1	65.70 ± 18.89	68 (26)	90.87 ± 42.16	85 (86)
*Z* (*p*)				−5.213 (< 0.001)**		−1.838 (0.066)	
Income level	Less than 60.000	171	36.0	65.96 ± 16.54	68 (16)	82.12 ± 33.93	74 (57)
	60.000 and over	304	64.0	83.97 ± 18.63	74 (32)	110.08 ± 35.71	123 (58)
*Z* (*p*)				−8.446 (< 0.001)**		−7.457 (< 0.001)**	
Number of children	^A^ One	193	40.6	77.56 ± 25.00	73 (41)	96.40 ± 38.12	91 (91)
	^B^ Two	183	38.5	77.89 ± 17.04	74 (25)	106.49 ± 36.43	120 (69)
	^C^ Three or more	99	20.8	76.62 ± 12.06	75 (2)	95.07 ± 37.03	109 (60)
*H* (*p*)				1.578 (0.454)		9.309 (0.010)*	
*Bonferroni correction*						A < (B = C)	
Chronic illness	Yes	146	30.7	82.39 ± 18.76	74 (32)	118.58 ± 30.61	123 (23)
	No	329	69.3	75.32 ± 20.00	74 (29)	91.77 ± 37.41	85 (82)
*Z* (*p*)				−3.285 (0.001)*		−6.510 (< 0.001)**	

*Z* Mann Whitney U Test, *H* Kruskal Wallis H Test, *n* sayı, *%* frekans, *SD* Standard Deviation, *M* Median, *IQR* Interquartile Range, *MPPS* Multidimensional Parenting Perfectionism Scale, *CSS* Cyberchondria Severity Scale

^*^p < 0.05, **p < 0.001

Table 4 presents the results of the multiple linear regression analysis conducted to identify the predictors of parental perfectionism. The regression model, which included the variables of age, educational attainment, employment status, income level, presence of chronic illness and severity of cyberchondria, was found to be statistically significant (F = 89.470, *p* < 0.001). The model explains 57.3 per cent of the total variance in parental perfectionism (R^2^ = 0.573). An examination of the regression coefficients revealed that the age variable was not a significant predictor of parental perfectionism (β = 0.093, *p* = 0.429). In contrast, educational attainment (β = 3.115, 95% CI [0.317, 5.913], *p* = 0.029), employment status (β = 4.680, 95% CI [0.773, 8.586], *p* = 0.019), income level (β = 7.328, 95% CI [3.724, 10.931], *p* < 0.001), presence of chronic illness (β = −6.296, 95% CI [−8.995, −3.597], *p* < 0.001) and severity of cyberchondria (β = 0.363, 95% CI [0.330, 0.396], *p* < 0.001) significantly predicted parental perfectionism. When the effects of other variables were controlled for, it was found that participants with a postgraduate level of education had higher scores for parental perfectionism, that employed participants exhibited higher levels of parental perfectionism compared to those not in employment, and that a high income level was associated with an increase in parental perfectionism. It was also found that participants without a chronic illness had lower parenting perfectionism scores than those with a chronic illness. It is noteworthy that the strongest predictor in the model was the severity of cyberchondria. Accordingly, a one-unit increase in the severity of cyberchondria was found to be associated with an increase of approximately 0.36 units in the parenting perfectionism score. In the analysis of the model assumptions, it was found that the Durbin–Watson statistic was 2.010, with tolerance values ranging from 0.552 to 0.911 and VIF values ranging from 1.098 to 1.811. These findings indicate that there are no problems of autocorrelation or multicollinearity in the model. Furthermore, the White test for heteroscedasticity was found to be significant (χ^2^ = 95.713, *p* < 0.001) and, consequently, the regression coefficients were reported using robust standard errors (Table 4).

**Table 4:** Multiple linear regression analysis to determine the effect of demographic characteristics and the severity of cyberchondria on parenting perfectionism (*n* = 475)

	*β*	*Robust se*	*t*	*p*	*β* for %95 *CI*	
					*Lower bound*	*Upper bound*
Model: MPPS						
Fixed	19.540	4.692	4.165	< 0.001**	10.320	28.760
Age	0.093	0.118	0.791	0.429	−0.139	0.326
Level of education	3.115	1.424	2.188	0.029*	0.317	5.913
Employment status (***Ref:*** Working)	4.680	1.988	2.354	0.019*	0.773	8.586
Income level	7.328	1.834	3.996	< 0.001**	3.724	10.931
Chronic illness (Ref: Yes)	−6.296	1.374	−4.584	< 0.001**	−8.995	−3.597
CSS	0.363	0.017	21.714	< 0.001**	0.330	0.396
Statistical Significance of the Model: *F* = 89.470; *p* < 0.001; *R*^2^ = 0.573						
White Test for Heteroskedasticity: *χ*^2^ = 95.713; *p* < 0.001						

Dependent Variable: MMP; Durbin-Watson: 2.010; Tolerance: 0.552 ~ 0.911; Variance inflation factor: 1.098 ~ 1.811

*β* unstandardized coefficients, *se* standard error, *CI* confidence interval

^*^p < 0.05, **p < 0.001

A post-hoc power analysis was conducted using G*Power 3.1.9.7 software to assess the explanatory power of the variables influencing MPPS scores. The analysis was based on a multiple linear regression model. The coefficient of determination (R^2^) for the model obtained in the study was found to be 0.573. The effect size calculated from this value was determined to be f^2^ = 1.34 according to the formula proposed by Cohen [109]. The threshold values proposed by Cohen [109] for f^2^ are 0.02 (small effect), 0.15 (medium effect) and 0.35 (large effect). Accordingly, the effect size obtained corresponds to a very large effect level. When this effect size was evaluated taking into account the sample size (*n* = 475), the significance level (α = 0.05) and the number of predictors in the model, the study’s statistical power was determined to be approximately 99.9%.

Before proceeding to the regulatory effect analysis, a multiple linear regression analysis was carried out to identify the socio-demographic variables influencing parental perfectionism. The results of the analysis showed that educational attainment, employment status, income level and the presence of chronic illness were significant predictors of parental perfectionism (*p* < 0.05). For this reason, these variables were controlled for as covariates in the moderational models. To test the moderating role of demographic variables on the effect of cyberchondria severity on parental perfectionism, analyses based on 5,000 bootstrap samples were conducted using the Hayes PROCESS macro (Model 1). In all models, employment status (1 = Working) and chronic illness status (1 = Yes) were included in the analyses using reference coding. The findings of the analyses showed that the effect of cyberchondria severity on parental perfectionism was significant in all models (*p* < 0.05). The values of explained variance for the models ranged from R^2^ = 0.573 to 0.584. However, the findings regarding moderating effects vary. The moderating effect of educational level was found to be statistically significant (β = 0.080, *p* = 0.016). This finding indicates that as educational level increases, the effect of cyberchondria severity on parental perfectionism becomes stronger. However, it was determined that the variance explained in this model showed only a limited increase (ΔR^2^ = 0.005). Similarly, income level was also found to be a significant moderating variable (β = 0.134, *p* < 0.001). The results indicate that as income level increases, the relationship between the severity of cyberchondria and parental perfectionism becomes stronger. The increase in explained variance in this model is ΔR^2^ = 0.011. In contrast, it was determined that the variables “employment status” (β = 0.056, *p* = 0.199) and “chronic illness status” (β = 0.017, *p* = 0.682) do not moderate the relationship between the severity of cyberchondria and parenting perfectionism. Overall, the findings suggest that the effect of cyberchondria severity on parenting perfectionism is partially mediated by socio-economic variables (particularly educational attainment and income level), whilst health status and employment status do not play a significant moderating role in this relationship (Table 5). In addition, the fit indices CMIN(χ^2^/df), RMSEA, SRMR, IFI, TLI, CFI, and GFI were examined to assess the model’s factor validity. The analysis revealed that the model’s fit indices fell within acceptable limits. Accordingly, the CMIN(χ^2^/df) value was calculated as 3.423, indicating that the model had an acceptable level of fit. The RMSEA value was found to be 0.071, the SRMR value 0.046, the IFI value 0.999, the CFI value 0.999, the GFI value 0.994, and the TLI value 0.984. It is noted that the RMSEA index is relatively less affected by sample size and that a range of 0.06–0.08 is generally considered an acceptable range in the literature. Furthermore, IFI, CFI, GFI, and TLI values above 0.90 indicate good model fit. In this study, the following criteria were used for the fit indices: RMSEA ≤ 0.08, IFI, CFI, and TLI ≥ 0.90, and GFI ≥ 0.85. The findings indicate that the model demonstrates generally acceptable fit and that the structural model has been validated.

**Table 5:** The moderating role of demographic characteristics in the effect of cyberchondria severity on parental perfectionism (*n* = 475)

		**Moderating Variables**							
		Level of education		Employment status (***Ref:*** Working)		Income level		Chronic illness (**Ref:** Yes)	
		***β* ± *se***	***p***	***β* ± *se***	***p***	***β* ± *se***	***p***	***β* ± *se***	***p***
Fixed		33.562 ± 5.235	<.001**	26.839 ± 4.206	<.001**	41.787 ± 5.972	<.001**	23.157 ± 2.934	<.001**
CSS		0.242 ± 0.052	<.001**	0.317 ± 0.038	<.001**	0.141 ± 0.064	.028*	0.356 ± 0.020	<.001**
Control Variables	Level of education	−5.181 ± 3.767	.170	3.333 ± 1.389	.017*	3.455 ± 1.374	.012*	3.221 ± 1.400	.022*
	Employment status (***Ref:*** Working)	4.761 ± 1.952	.015*	−0.234 ± 4.373	0,957	3.732 ± 1.96	0,058	4.686 ± 1.980	.018*
	Income level	8.377 ± 1.720	<.001**	7.174 ± 1.682	<.001**	−4.158 ± 3.627	0,252	7.381 ± 1.679	<.001**
	Chronic illness (**Ref:** Yes)	−6.824 ± 1.477	<.001**	−6.521 ± 1.483	<.001*	−7.569 ± 1.494	<.001**	−8.086 ± 4.855	.096
Model Significance (Total Effect)		*R*^*2*^** = **0.578		*R*^*2*^ **= **0.575		*R*^*2*^** = **0.584		*R*^*2*^ **= **0.573	
		*F* = 106.617 *p* < 0.001		*F* = 104.985 *p* < 0.001		*F* = 109.307 *p* < 0.001		*F* = 104.407 *p* < 0.001	
Interaction effect		0.080 ± 0.033	0.016	0.056 ± 0.043	0.199	0.134 ± 0.037	< 0.001**	0.017 ± 0.041	0.682
Model Significance of the Moderation Effect		*R*^*2*^** = **0.005		*R*^*2*^ **= **0.002		*R*^*2*^** = **0.011		*R*^*2*^ **= **0.000	
		*F* = 5.839 *p* **= **.016		*F* = 1.652 *p*** = **.199		*F* = 12.743 *p* < 0.001		*F* = 0.168 *p* = 0.682	

*β* unstandardized coefficients, *se* standard error

^*^p < 0.05, **p < 0.001

## Discussion

This study examines the relationship between cyberchondria tendencies and parenting perfectionism among mothers aged 25–50 with children in early childhood; it was conducted using a purposive sample of mothers who met the criteria of voluntary participation, sufficient Turkish language proficiency, and the absence of a psychiatric diagnosis.

The research findings revealed a statistically significant, positive correlation between parents’ overall levels of cyberchondria severity and their levels of parenting perfectionism. This finding suggests that online health information-seeking behavior is not merely a functional process aimed at acquiring information; it may also reflect cognitive processes related to parents’ perceptions of responsibility for their children’s health, their tendency to avoid making mistakes, and the high parenting standards they set for themselves. In particular, the abundance of often ambiguous health information encountered online in a parenting context may lead parents to feel a need to make flawless decisions regarding their children’s health. This situation may reinforce parents’ perfectionist expectations of themselves while they engage in repeated information-seeking behaviors aimed at reducing health-related uncertainty. This interpretation is consistent with previous studies highlighting the relationship between health anxiety, obsessive beliefs, and perfectionism. Karadayı [110] reported that health anxiety and obsessive beliefs are associated with perfectionism; Sapmaz [111] noted that obsessive–compulsive tendencies and anxiety symptoms are observed at higher levels in perfectionistic individuals. Similarly, it has been noted that cyberchondria is fueled not only by health anxiety but also by repetitive and compulsive checking behaviors aimed at eliminating uncertainty [99, 112, 113]. These common psychological mechanisms can be considered one of the possible explanations underlying the relationship between cyberchondria and parental perfectionism.

The research findings indicate that parenting perfectionism varies significantly by age, but the severity of cyberchondria is not associated with age. Accordingly, the higher levels of parenting perfectionism observed among mothers aged 35 and older can be explained by the fact that responsibilities related to the parenting role become more central and defining over time. As parenting experience increases, the perception of responsibility regarding children’s developmental processes strengthens, and parents may tend to evaluate their own performance against higher standards. This situation may pave the way for the emergence of a desire to avoid making mistakes in the parenting context and stricter self-evaluation processes. Although studies directly examining the relationship between parenting perfectionism and age are limited in the literature, it is noted that perfectionism is a dynamic construct shaped by interactions with life roles, social expectations, and contextual responsibilities, rather than a static personality trait [114]. Similarly, Sand et al.118 have emphasized that perfectionism can vary depending on sociodemographic and environmental variables. In this context, the current finding suggests that parental perfectionism may intensify alongside changing parenting demands and a growing sense of responsibility throughout the life course. In contrast, the fact that the severity of cyberchondria does not vary by age group suggests that the behavior of searching for health information online has become a widespread pattern observed at similar levels across different age groups today. The current finding is consistent with the results reported by Barke et al. [77] and Vismara et al. [66], which also revealed no significant association between age and cyberchondria. The fact that age did not emerge as a significant predictor in either study suggests that cyberchondria may be a construct related to individuals’ cognitive evaluations of health and their emotional responses to uncertainty, rather than demographic characteristics. In this context, it is believed that cyberchondria may be more strongly explained by age-independent psychological mechanisms, such as health anxiety, intolerance of uncertainty, and a tendency to seek reassurance.

The research findings indicate that educational level significantly predicts parental perfectionism but has no significant effect on the severity of cyberchondria. The fact that mothers with graduate-level education exhibit higher levels of parenting perfectionism can be explained by the increased achievement orientation, self-evaluation tendencies, and higher standards regarding the parenting role that come with education. Indeed, increased socioeconomic and educational resources not only enable parents to access more information about child development but may also raise their expectations regarding child-rearing [115, 116]. This situation may lead parents with higher levels of education to perceive greater responsibility for their children’s developmental processes, prompting them to evaluate their parenting performance within a more critical framework. Conversely, the fact that the severity of cyberchondria does not vary by educational level can be explained by the fact that while education increases access to online health information, it also develops individuals’ ability to evaluate and question this information. In other words, while a higher level of education may increase health-related information-seeking behavior, it can prevent health anxiety from rising to excessive levels thanks to critical thinking and information verification skills.

The study found that working mothers have higher levels of parental perfectionism than non-working mothers. This finding can be explained by the fact that working mothers must simultaneously meet expectations to succeed in both their professional and parenting roles. The literature also indicates a positive relationship between the pressure to be a “perfect mother” and women’s career goals [117]. Furthermore, societal norms that raise expectations regarding ideal motherhood [118] may reinforce working mothers’ efforts to maintain high standards in both their professional and parenting roles [119]. However, the fact that employment status does not significantly influence the severity of cyberchondria suggests that the behavior of searching for health information online has become a widespread pattern of behavior today, independent of employment status.

The research findings indicate that income level has a significant effect on both parenting perfectionism and the severity of cyberchondria. Accordingly, mothers in the high-income group were found to have higher levels of both parenting perfectionism and cyberchondria. In terms of parenting perfectionism, this result can be explained by the fact that parents with high socioeconomic resources are able to invest more in their children’s developmental, academic, and social achievements and, consequently, evaluate their parenting roles against higher standards. While increased resources enable parents to access more information, activities, and opportunities to support their children’s development, they can also raise expectations regarding the parenting role, thereby increasing self-critical evaluations and performance pressure. Indeed, Sand et al. [115] and Wang et al. [116] have reported that socioeconomic status is associated with perfectionist tendencies. However, the current finding differs somewhat from the results reported by Wang et al. [116]. In that study, it was noted that child-oriented perfectionism in families with low socioeconomic status may function as a compensatory coping strategy in the face of economic stress and limited emotion regulation skills. In contrast, the finding in the present study that parenting perfectionism was higher among mothers with higher income levels suggests that the relationship between socioeconomic status and perfectionism is not linear. While perfectionism may emerge as a compensatory mechanism for coping with stress in low socioeconomic conditions, increased parental investment, expectations of success, and a sense of responsibility in high socioeconomic conditions may reinforce perfectionist tendencies. This suggests that the relationship between socioeconomic conditions and parental perfectionism may be influenced by factors such as cultural context and parenting norms. From a cyberchondria perspective, a high income level may be associated with easier access to digital health resources and an increased search for information regarding children’s health. This situation may contribute to the persistence of cyberchondria symptoms by supporting repetitive reassurance-seeking behaviors aimed at reducing health-related uncertainties. Therefore, the effect of income level on cyberchondria should be evaluated in conjunction with parents’ perceptions of health responsibility and their tendencies to seek reassurance, rather than solely in terms of economic resources.

The research findings show that the number of children does not make a significant difference in parenting perfectionism; however, the severity of cyberchondria increases in proportion to the number of children. The fact that parenting perfectionism is independent of the number of children suggests that this construct is related more to parents’ standards for themselves, their expectations of success, and their cognitive evaluation processes than to the quantitative burden of parenting. In other words, how parents perceive their parenting role and their expectations regarding this role appear to be more influential in determining their level of perfectionism than the number of children they have. Conversely, the association between an increase in the number of children and the severity of cyberchondria can be explained by the increase in parenting responsibilities and uncertainties regarding the children’s health status. As the number of children increases, the health issues, developmental processes, and care needs that parents may encounter also diversify; this situation can heighten perceptions of health-related risks and the need for control. The growing search for control and reassurance, in turn, can lead parents to consult online health information more frequently and, over time, cause this behavior to become a repetitive pattern. Indeed, the current finding is consistent with previous studies reporting that levels of cyberchondria rise as the number of children increases [84, 120, 121]. This finding suggests that an increase in the number of children for whom parents are responsible may intensify their efforts to manage health-related uncertainties and their behavior in seeking health information online.

Research findings have shown that mothers with chronic illnesses have significantly higher scores on both parenting perfectionism and cyberchondria severity scales compared to mothers without chronic illnesses. This can be explained by the fact that chronic illness increases individuals’ perception of health-related threats and necessitates more intensive monitoring of their health status in daily life. The need for constant health monitoring may cause individuals to become more sensitive to health-related uncertainties and to consult online health information more frequently. When evaluated specifically in the context of parenting, the combination of concerns about one’s own health with responsibilities for children’s health and safety can lead parents to elevate both their health-related control behaviors and their parenting standards. Therefore, it can be expected that mothers with chronic illnesses will not only engage in more intensive searches for online health information but also exhibit stronger perfectionist tendencies aimed at avoiding mistakes in their parenting roles. When evaluated in terms of cyberchondria, the current finding is consistent with studies reporting higher levels of cyberchondria among individuals with chronic illnesses [84, 122]. However, there are also studies in the literature reporting that individuals with chronic illnesses have lower levels of cyberchondria [123], that chronic illness does not affect cyberchondria [124], or that lower levels of cyberchondria were observed in individuals without chronic illnesses [125]. These differing findings indicate that the presence of a chronic illness alone is not a determining factor for cyberchondria; rather, it must be evaluated in interaction with various variables such as health anxiety, intolerance of uncertainty, the type and severity of the illness, and access to healthcare services. In the current study, the higher severity of cyberchondria found among mothers with chronic illnesses may be related to these individuals being more sensitive to symptom monitoring and turning to online resources more frequently to reduce health-related uncertainty. Conversely, the finding in some studies that individuals with chronic illness exhibit lower levels of cyberchondria may be explained by their regular contact with healthcare professionals and their development, over time, of more realistic assessments of their health status, thereby gaining a higher tolerance for uncertainty. Therefore, it can be said that the relationship between chronic illness and cyberchondria is not linear but rather a multidimensional structure shaped by the influence of individual and contextual factors.

The results of the regression analysis showed that the severity of cyberchondria is a significant predictor of parental perfectionism. This finding suggests that online health information-seeking behaviors are not limited to the purpose of acquiring information, but may also be linked to cognitive and emotional processes related to parenting standards. It is hypothesized that online information-seeking behaviors, which intensify particularly in situations of health-related uncertainty, may emerge in conjunction with parents’ perfectionist tendencies to avoid making mistakes and make the most accurate decisions—tendencies associated with intolerance of uncertainty and a need for control [126, 127]. Within this framework, cyberchondria can be interpreted as an important psychological process that is interrelated with parental perfectionism and may be associated with the maintenance of parenting standards.

Moderator analyses have shown that education and income level significantly strengthen the relationship between the severity of cyberchondria and parenting perfectionism. Accordingly, as education and income levels rise, the relationship between cyberchondria and parenting perfectionism becomes more pronounced. This finding can be explained by the fact that parents with a high socioeconomic status not only have easier access to online health information but also hold higher expectations and standards regarding child-rearing. In contrast, the lack of a significant moderating effect for the variables of employment status and chronic illness suggests that these variables may indicate independent or direct relationships rather than modifying the relationship in question. Overall, these findings indicate that the relationship between cyberchondria and parenting perfectionism does not manifest at the same level among all parents; the socioeconomic context is a key factor shaping the strength of this relationship. In this regard, the study indicates that the relationship between cyberchondria and parenting perfectionism must be evaluated by taking into account not only individual psychological characteristics but also socioeconomic factors.

### Theoretical ımplications

This study contributes to the literature at a theoretical level by focusing on the relationship between cyberchondria and parental perfectionism. The findings suggest that this relationship should be assessed not only in the context of individual health anxiety, but also within the framework of cognitive processes that unfold in digital information environments. Cyberchondria may be associated with an increased perception of uncertainty linked to online health information and a concomitant rise in the need for control [68]. Parents’ online health information-seeking behaviors for their children have been shown to be frequent and often driven by concerns about uncertainty and reassurance needs [89], which may be conceptually consistent with perfectionistic parenting patterns such as high standards and low tolerance for mistakes. Overall, the findings suggest that digital information environments must be taken into account when explaining parenting perfectionism. In this context, cyberchondria can be regarded as a digital-based risk indicator that may influence parenting cognitions.

### Practical ımplications

The findings suggest that the relationship between cyberchondria and parenting perfectionism may be an area worth considering in intervention studies. Given that a significant proportion of parenting decisions today are linked to online health information, it is important for interventions to focus not only on parenting skills but also on the relationship with digital information. At the individual level, interventions aimed at developing digital health literacy are becoming increasingly important [86–88]. Strengthening parents’ ability to critically evaluate online health information, question the reliability of sources and verify information may help to reduce the cognitive cycles associated with cyberchondria. In addition, cognitive-behavioural approaches that support skills for coping with uncertainty can help to reduce control-oriented thought patterns. At the family level, it is recommended that parenting education programmes address not only child development and discipline processes, but also the relationship with digital information. Awareness-raising initiatives targeting perfectionist parenting tendencies can help foster more flexible and realistic parenting expectations. Psycho-educational interventions, meanwhile, can support the development of a more balanced cognitive framework by targeting factors such as health anxiety, intolerance of uncertainty and excessive reliance on digital information. Overall, the findings suggest that the focus of intervention may lie not only in reducing cyberchondria but also in strengthening digital cognitive regulation skills.

## Implications for future research

It is important for future research to examine the relationship between cyberchondria and parental perfectionism using more robust methodological designs. First and foremost, longitudinal studies are essential for elucidating the nature and changes in this relationship over time. Such research could provide clearer answers to the question of whether cyberchondria increases parenting perfectionism or whether perfectionist tendencies create a predisposition to cyberchondria. It will also contribute to understanding the trajectory of these variables throughout the stages of child development. In addition, it is recommended that experimental and quasi-experimental studies be increased. Studies in which the level of exposure to online health information is manipulated may offer the opportunity to test the causal effects of increases in cyberchondria on parenting cognitions and behaviours. Such studies will also be important in identifying intervention points for digital health anxiety. Furthermore, future research must increase sample diversity. In particular, the greater inclusion of fathers in studies is important for understanding gender-based differences in parenting processes. The fact that the existing literature is largely mother-centred limits the generalisability of the findings. However, comparative studies conducted across different cultural contexts may reveal the culture-dependent variability of digital health behaviours and parenting perfectionism. Similarly, the inclusion of different age groups of children (such as early childhood, pre-school and primary school) would better elucidate the role of developmental differences in this relationship. Finally, it is recommended that future research develop more comprehensive models incorporating mediating variables. In particular, it is thought that variables such as health anxiety, intolerance of uncertainty, parental self-efficacy and digital health literacy may play a role as important psychological and cognitive mechanisms in this relationship. Including these variables in the models will contribute to a more explanatory and theoretically robust examination of the relationship between cyberchondria and parental perfectionism.

## Limitations and methodological considerations

When interpreting the findings of this study, certain methodological and conceptual limitations must be taken into account. Firstly, the fact that the study employs a cross-sectional design and that data were collected at a single point in time using self-report measures limits the ability to interpret the relationships between variables as causal. Furthermore, the fact that all variables were obtained from the same participants using the same measurement method carries the risk of common method variance and raises the possibility that some of the observed relationships may appear stronger than they actually are due to methodological factors. For this reason, the relationships between variables should be assessed solely at a statistical level, and causal inferences should be avoided. Whilst it has been found that the severity of cyberchondria predicts parental perfectionism, the presence of reverse or reciprocal effects is also possible. It is thought that future longitudinal and experimental studies will contribute both to a clearer understanding of causal mechanisms and to the acquisition of stronger evidence with common-method bias controlled for. Secondly, the research sample consists solely of mothers with children in early childhood and was selected using a purposive sampling method. Therefore, care should be taken when generalising the findings to different groups of parents, carers or populations with different socio-cultural characteristics. In addition, the fact that the sample consists largely of mothers with high levels of education, who are employed, and who belong to a relatively high-income group limits the generalizability of the findings to populations with different socioeconomic statuses in terms of the study’s external validity. Thirdly, although the internal consistency coefficients of the scales used in the study were found to be high, confirmatory factor analysis was not carried out on the current sample. Whilst validity and reliability studies of the Turkish adaptations of the measurement instruments have been conducted previously, re-confirming the factor structure in different samples could strengthen the evidence regarding the construct validity of the measurements. Fourthly, although the multiple linear regression model explained a significant proportion of the variance in parental perfectionism, the presence of unexplained variance indicates that parental perfectionism cannot be explained solely by cyberchondria and the socio-demographic variables examined in the study. In particular, it is recommended that variables such as health anxiety, intolerance of uncertainty, parental self-efficacy, digital health literacy, perceived social support and psychological well-being be included in the model in future research. Fifthly, in this study, the moderating effects of demographic variables were examined using the bootstrap-based PROCESS macro. Whilst this approach offers a robust and suitable method for testing research hypotheses, given the multidimensional structures of cyberchondria and parenting perfectionism, the use of structural equation modelling and latent variable approaches in future studies may contribute to a more comprehensive assessment of the relationships between sub-dimensions and measurement errors. Sixthly, there are some limitations in the literature regarding the psychometric structure of the “Distrust of Healthcare Professionals” sub-dimension. The fact that the items in this sub-dimension encompass both the hierarchy of trust (doctor vs. the internet) and information-related emotional regulation processes may lead to conceptual heterogeneity. This is considered a factor that could explain the relatively low internal consistency coefficient. Furthermore, previous studies [112, 128] have reported that this sub-dimension may exhibit weaker psychometric properties compared to other dimensions, and in some cases, its removal from the scale has been recommended. In addition, it is thought that cultural context may also play a role in the functioning of this sub-dimension. In contexts where relatively high trust in medical authority coexists with online health information-seeking behaviour, the fact that participants’ responses reflect situational and variable patterns of trust rather than a fixed latent structure may be another factor that could weaken internal consistency. For this reason, no analysis was conducted at the sub-dimension level in the present study; evaluation was carried out solely on the basis of the total scale score. Finally, the study found that the contributions of the moderating effects identified in the study to the explained variance were limited. This highlights that the relationship between parental perfectionism and cyberchondria cannot be explained by demographic characteristics alone, and that other psychological and contextual mechanisms underlying this relationship need to be addressed. It is thought that holistic models developed along these lines could provide stronger theoretical and empirical evidence to explain the relationship between parental perfectionism and digital health behaviours.

## Conclusion

This study examines the relationship between cyberchondria and parenting perfectionism, revealing a significant and positive correlation between the two variables. The findings suggest that as parents’ online health information-seeking behaviour increases, their tendencies towards perfectionist parenting may also intensify. This suggests that the constant monitoring of health and the search for information in the digital environment may foster a parenting pattern characterised by greater control, low tolerance for error and high standards in the child-rearing process. The analysis results also reveal that cyberchondria is a significant and powerful predictor of parenting perfectionism. This finding demonstrates that digital health anxiety should be considered not merely as an individual pattern of anxiety, but also as a fundamental psychological mechanism that shapes parenting attitudes and behaviours. In particular, the compulsive nature of searching for health information online can reduce parents’ tolerance for uncertainty, thereby triggering more rigid and control-oriented parenting approaches. However, it has been observed that socio-demographic variables such as educational level and income play a moderating role in this relationship. It is understood that the relationship between cyberchondria and perfectionism differs among parents with higher levels of education and income; ease of access to digital information and increased health literacy can both reinforce this dynamic and, under certain conditions, counterbalance it. This finding highlights the indirect effects of digital inequalities and socio-economic disparities on parenting processes. Consequently, this study makes a significant contribution to the literature on digital parenting. By revealing the impact of cyberchondria on parenting perfectionism, it expands the role of digital health anxiety within the psychology of parenting. The findings emphasise the need for future studies to address psychological (e.g. intolerance of uncertainty, health anxiety) and contextual (e.g. digital ecosystem, socio-economic structure) variables within more holistic models.

## Data Availability

The datasets generated during and/or analysed during the current study are available from the corresponding author upon reasonable request.
